# Electron streaming dose measurements and calculations on a 1.5 T MR‐Linac

**DOI:** 10.1002/acm2.14370

**Published:** 2024-04-25

**Authors:** Elizabeth Patterson, Marcus Powers, Peter E. Metcalfe, Dean Cutajar, Bradley M. Oborn, John A. Baines

**Affiliations:** ^1^ Centre for Medical and Radiation Physics University of Wollongong Wollongong New South Wales Australia; ^2^ College of Science and Engineering James Cook University Townsville Queensland Australia; ^3^ Townsville Cancer Centre Townsville Hospital and Health Service Townsville Queensland Australia; ^4^ Illawarra Health Medical Research Institute University of Wollongong Wollongong New South Wales Australia; ^5^ Department of Radiation Oncology St George Cancer Care Centre Wollongong New South Wales Australia; ^6^ Institute of Radiooncology‐ OncoRay Helmholtz‐Zentrum Dresden‐Rossendorf, Radiooncology Dresden Germany; ^7^ Illawarra Cancer Care Centre Wollongong Hospital Wollongong New South Wales Australia

**Keywords:** electron streaming effect, Elekta Unity, Monaco Unity, MRgRT, MR‐linac, out‐of‐field dose, skin dose

## Abstract

**Purpose:**

To evaluate the accuracy of different dosimeters and the treatment planning system (TPS) for assessing the skin dose due to the electron streaming effect (ESE) on a 1.5 T magnetic resonance (MR)‐linac.

**Method:**

Skin dose due to the ESE on an MR‐linac (Unity, Elekta) was investigated using a solid water phantom rotated 45° in the x‐y plane (IEC61217) and centered at the isocenter. The phantom was irradiated with 1 × 1, 3 × 3, 5 × 5, 10 × 10, and 22 × 22 cm^2^ fields, gantry at 90°. Out‐of‐field doses (OFDs) deposited by electron streams generated at the entry and exit surface of the angled phantom were measured on the surface of solid water slabs placed ±20.0 cm from the isocenter along the x‐direction. A high‐resolution MO*Skin*™ detector served as a benchmark due to its shallower depth of measurement that matches the International Commission on Radiological Protection (ICRP) recommended depth for skin dose assessment (0.07 mm). MO*Skin*™ doses were compared to EBT3 film, OSLDs, a diamond detector, and the TPS where the experimental setup was modeled using two separate calculation parameters settings: a 0.1 cm dose grid with 0.2% statistical uncertainty (0.1 cm, 0.2%) and a 0.2 cm dose grid with 3.0% statistical uncertainty (0.2 cm, 3.0%).

**Results:**

OSLD, film, the 0.1 cm, 0.2%, and 0.2 cm, 3.0% TPS ESE doses, underestimated skin doses measured by the MO*Skin*™ by as much as –75.3%, –7.0%, –24.7%, and –41.9%, respectively. Film results were most similar to MO*Skin*™ skin dose measurements.

**Conclusions:**

These results show that electron streams can deposit significant doses outside the primary field and that dosimeter choice and TPS calculation settings greatly influence the reported readings. Due to the steep dose gradient of the ESE, EBT3 film remains the choice for accurate skin dose assessment in this challenging environment.

## 1 INTRODUCTION

Online magnetic resonance (MR)‐guided adaptive radiotherapy (MRgART) enables real‐time soft‐tissue visualization to monitor anatomy motion and to adapt treatment plans accordingly. Hybrid systems that integrate magnetic resonance imaging (MRI) and megavoltage (MV) linear accelerator (linac) technology are commonly referred to as MR‐linac or MRI‐linac systems. The presence of the static magnetic field (B_0_) in an MR‐linac is known to affect the radiation beam generation and the transport of charged particles to and within the patient.^1^ Systems where the MV beam is delivered perpendicular to B_0,_ also known as transverse MR‐linacs, are of interest in this investigation. To date, two commercial MR‐linac systems have been developed and are currently available for clinical use.^2^, ^3^

The magnetic field present in MR‐linacs deflects secondary electrons generated within the system components,^4^ air,^5^ imaging coils, immobilization devices,^6^, ^7^ and the patient,^8^ depending on their initial trajectory relative to the B_0_ direction. For transverse MR‐linac systems, electrons that travel perpendicular to B_0_ experience circular motion about the field lines,^9^ while those with parallel and perpendicular components of velocity, spiral helically about B_0_ lines.^5^, ^8^ In standard non‐MR‐linac systems, the flattening filter, if present, serves as a primary source of contaminant electrons. For a transverse configured MR‐linac, these electrons will not reach the patient surface due to interactions with the B_0_. Charged particle transport in longitudinal magnetic fields, B_0_ parallel to the MV beam, has been described in detail elsewhere.^10^ These systems are commonly known as inline MR‐linacs.

The magnetic field in a transverse MR‐linac causes dose perturbation effects including a lateral shift in the dose distribution and penumbra asymmetry,^11^ a reduction in the depth of maximum dose deposited (d_max_),^11^ the electron return effect (ERE),^9^ spiraling contaminant electrons (SCE),^5^, ^6^, ^12^ and the electron streaming effect (ESE).^7^, ^8^, ^13^, ^14^, ^15^, ^16^ Contaminant electrons generated by the irradiation of air away from the linac head can spiral along the B_0_ lines and are commonly known and referred to as SCE.^5^, ^12^ ESE is associated with electron streams from backscattered or forward‐ejected electrons, originating from beam interactions within scattering objects (i.e., patients, phantoms, imaging coils, or immobilization devices).^6^, ^7^, ^8^ Typically, SCE produces a smaller out‐of‐field dose (OFD) than ESE.^12^, ^13^

In a transverse MR‐linac, approximately 0.5 MeV secondary electrons^17^ with a continuous slowing down approximation (CSDA) range of approximately 1.5 m in air, can form electron streams that deposit dose far from the treatment fields in the cranio‐caudal direction.^6^ Clinical observations have confirmed the occurrence of OFDs from electron streams,^6^, ^7^, ^8^, ^15^, ^16^, ^18^ which possess the potential to cause skin injuries, including erythema, as a result of unintended and non‐therapeutic irradiation of the skin outside the radiation field.

In a multi‐patient accelerated partial‐breast irradiation (PBI) study, a prescription dose of 38.5 Gy was delivered across 10 fractions using a Co‐60 source ViewRay system (ViewRay, Oakwood Village, OH, USA). The investigation included in vivo film dosimetry which revealed an average OFD to the lower jaw of 0.54 Gy for a single fraction delivery across all the patients included in the study.^8^ Cumulatively across 10 fractions, a total of 5.4 Gy could have been delivered outside the treatment area if a 1.0 cm bolus intervention was not implemented.^8^ According to the AAPM TG‐158,^19^ this unshielded region would correspond to an intermediate out‐of‐field non‐target dose. Similarly, in vivo film dosimetry was performed for PBI with a prescription dose of 40.05 Gy on an Elekta Unity (Elekta, Stockholm, Sweden) system and measured a summed average dose to the chin of 2.69 Gy across 15 fractions. This dose is classified as an intermediate, although borderline low level, non‐target dose according to TG‐158.^15^ Summed across all fractions, this was reduced to 0.56 Gy using a 1.0 cm bolus placed at the chin, reducing the non‐target dose to a low dose level. Amongst the cohort of 11 patients treated on the MR‐linac with bolus placed at the chin, the authors reported no patients developed skin erythema at the chin.^15^ Film dosimetry used for a separate PBI treatment on an Elekta Unity measured a total of 2.6 Gy across 15 fractions to the chin which was reduced to 0.75 Gy with the introduction of bolus shielding, reducing the nontarget dose to the lowest dose level.^18^ For treatment of a supraclavicular lymph node with a prescription dose of 36.0 Gy delivered across four fractions, the treatment planning system (TPS) predicted a 9.9 Gy OFD to the ear.^6^ Although this dose is below the suggested skin dose constraint for ulceration in stereotactic body radiation therapy (SBRT) it could still lead to mild skin reactions without bolus shielding.^20^

Whilst these clinical studies demonstrate unwanted skin dose and consequently that skin reactions may occur outside the primary treatment field for MR‐linac treatments, appropriate shielding and patient setup can reduce the risk of skin reactions from electron streams.^6^, ^8^, ^15^, ^16^, ^18^ The use of bolus for shielding can be expedited if the planning images of the patient include regions at risk of exposure to electron streams. Furthermore, the TPS must account for air‐electron transport^21^ outside the patient to identify electron streams, enabling clinical staff to determine the appropriate shielding requirements before treatment delivery.

Experimental verification of electron stream OFD has primarily been conducted using film,^6^, ^7^, ^8^, ^14^, ^15^, ^18^, ^22^ and optically stimulated luminesce devices (OSLDs).^23^, ^24^ Clinical studies comparing film‐measured ESE doses to the TPS, generally found that the TPS overestimates the dose to out‐of‐field surfaces^15^, ^18^; however, the TPS has also been found to underestimate ESE dose originating from the anterior imaging coil on an Elekta Unity.^6^ Typically TPSs are not commissioned for OFD calculations and consequently, the accuracy of TPS‐determined OFD is poor. The AAPM TG 158 recommends that calculated doses by the TPS should not be relied upon beyond 3.0 cm from the field edges.^19^ Inaccurate OFD dose distributions predicted by the TPS can be attributed to an underestimation of patient scatter, head leakage, and collimator scatter.^25^ Verifying the accuracy of the TPS calculated electron streaming dose is relevant for optimal patient treatment and inconsistencies in the aforementioned studies warrant further investigation.

For non‐MR‐linac therapeutic photon beams, large dose gradients and electronic disequilibrium near the surface are known to limit skin dosimetry accuracy.^26^ Similarly, dose deposition of electron streams that can form in transverse MR‐linacs primarily occurs within the first few millimeters of tissue,^13^ which poses a challenge for accurate skin dosimetry. Therefore, thin detectors, capable of resolving doses within these regions, should be considered.^26^

This study aims to evaluate the suitability of different dosimeters and the TPS for assessing skin dose (i.e., at 0.07 mm depth), according to International Commission on Radiological Protection (ICRP) recommendations, from electron streams in a 1.5 T 7 MV MR‐linac. The MO*Skin*™, a metal‐oxide‐semiconductor field‐effect transistor (MOSFET) detector, was used in this study as it has been designed and benchmarked for skin dosimetry in megavoltage radiotherapy.^27^, ^28^, ^29^, ^30^, ^31^ Out‐of‐field MO*Skin*™ measurements taken at the surface of the phantom were compared to Gafchromic EBT3 film (Ashland ISP Advanced Materials, Wayne, NJ, USA), nanoDot OSLDs (Landauer, Glenwood, IL, USA), a PTW 60019 microDiamond (PTW, Freiburg, Germany), and the Monaco TPS (Elekta, Stockholm, Sweden), for multiple field sizes. To account for the inherent build‐up around the sensitive regions of these dosimeters and volume averaging across the dose voxels, MO*Skin*™ measurements were also performed at the approximate water‐equivalent‐depth (WED) corresponding to each dosimeter and the center of the dose scoring voxel size specified in the planning system. These measurements were conducted to offer additional insights by determining what the MO*Skin*™ reports at similar measurement locations.

Inside the primary radiation field, measurements conducted at depths greater than the ICRP recommended depth for skin dose assessment will lead to an overestimation of skin dose due to the steep dose gradient.^32^ Similarly, it is expected that the steep depth dose deposition of electron streams^13^ will largely impact dosimetry readings from different devices.

## 2 METHODS

All irradiations were performed on an Elekta Unity system which includes a 1.5 T MRI scanner (Philips Healthcare, Best, the Netherlands) combined with flattening‐filter‐free (FFF) 7 MV linac beam delivered at a direction perpendicular to B_0._^2,^ ^11^ The system has a source‐to‐axis distance (SAD) of 143.5 cm with a fixed couch height, located 14.0 cm below isocenter. The machine is calibrated isocentrically to give 1.00 cGy per MU, at a depth of 5.0 cm and a source‐to‐surface distance (SSD) of 138.5 cm, in a 10 × 10 cm^2^ field. At an SSD of 133.5 cm, 1.23 cGy per MU is delivered to d_max_ by a 10 × 10 cm^2^ beam.

### 2.1 MO*Skin*™ detector

The MO*Skin*™ can achieve excellent spatial resolution due to a small submicron dosimetric volume and 5.5 × 10^−4^ mm thickness of the sensitive sensor.^28^ The ICRP recommends a depth of 0.07 mm, corresponding to the average depth of the basal cell layer, for practical skin dose assessment.^27^ A thin, water‐equivalent polyamide film acts as a build‐up layer, giving the MO*Skin*™ a WED equal to 0.07 mm.^28^ In this work, the term “skin dose” is a clinical term referring to the dose at the recommended ICRP depth. Previous measurements with the MO*Skin*™ on the Elekta Unity^33^ and an inline 1.0 T MR‐linac^34^, ^35^, ^36^ indicate that the dose‐response of the detector is unaffected by the presence of the B_0_.^37^

#### 2.1.1 Calibration

Each MO*Skin*™ detector was positioned face‐up^28^ in a 1.0 cm‐thick, custom‐milled solid water phantom (Model number 457, Gammex/RMI, WI, USA) holder, with the detector centered at isocenter. A 5.0 cm thick Solid Water® HE (Gammex Inc—A Sun Nuclear Company, Middleton, WI, USA) block was used as build‐up and 10.0 cm as backscatter. Each MO*Skin*™ was irradiated with a 10 × 10 cm^2^, 100 MU, gantry 90° (G90) beam. For dose readout, each detector was connected to the OneTouch system.^38^ An average of three readouts were used to determine the calibration factor to convert mV to cGy.

#### 2.1.2 Measurements

**FIGURE 1:**
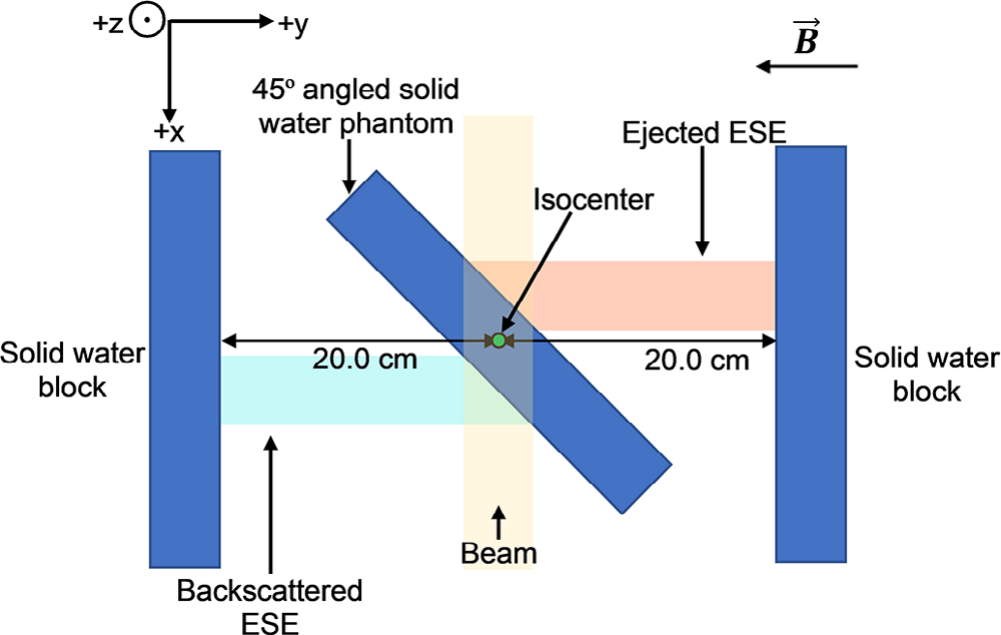
Schematic of the setup used to generate backscattered and ejected ESE.

Solid Water® HE blocks with dimensions of 30.0 × 30.0 × 5.0 cm^3^ were used for electron streaming measurements. Two blocks were positioned parallel to the x‐direction (IEC61217), ± 20.0 cm from the isocenter. A third block was angled at 45° relative to the y‐direction, with the center aligned to the isocenter, as shown in Figure 1. The angle of the third block was chosen to generate approximately square electron stream distributions when a square primary beam is applied. This setup aimed to assist in the reproducibility of each dosimeter's position, particularly in the x‐direction of the non‐uniform ESE dose distribution. For phantom setup reproducibility, the outline of each block was marked on the couch. A 1 × 1 cm^2^ G90 beam was delivered to the setup with RTQA2 film (Ashland ISP Advanced Materials, NJ, USA) placed on the outer solid water blocks, to locate optimal positions for the MO*Skin*™, OSLDs, and the microDiamond. Electron streams from 1 × 1 cm^2^, 3 × 3 cm^2^, 5 × 5 cm^2^, 10 × 10 cm^2^, and 22 × 22 cm^2^, G90, fields incident on the angled block were investigated. The dose from streaming backscattered electrons, generated as the beam enters the angled phantom, will be referred to as “backscattered ESE.” The dose from forward‐ejected electrons, produced from interactions at the beam exit from the phantom, will be referred to as “ejected ESE.”

**FIGURE 2:**
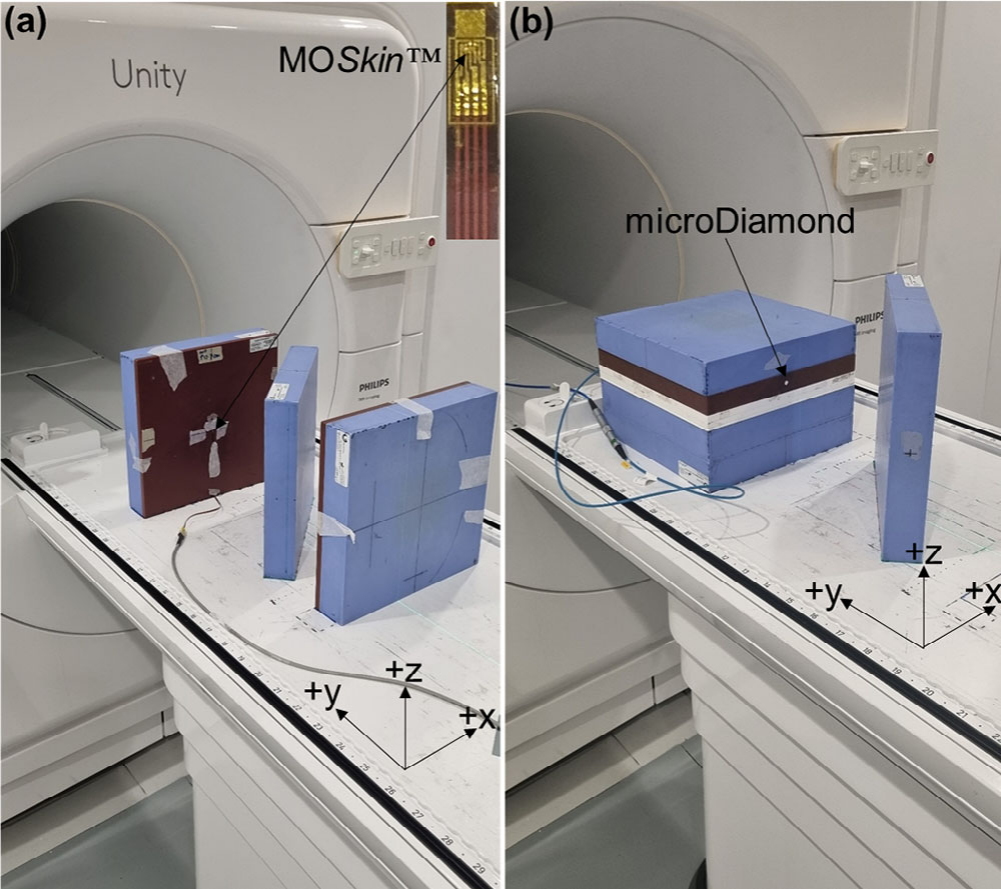
(a) Photograph of the experimental setup with the MOSkin™ positioned in the holder placed 20.0 cm from the isocenter to measure ejected ESE. (b) Photograph of the microDiamond positioned using a customized phantom to measure ejected ESE.

Two calibrated MO*Skin*™ detectors were used for electron streaming measurements. The detectors were placed in the 1.0 cm custom holder, positioned in front of each out‐of‐field solid water block (Figure 2a) at locations determined from the RTQA2 film measurements. When using the holder, the two 5.0 cm out‐of‐field blocks needed to be shifted 1.0 cm along the y‐direction to maintain a 20.0 cm distance from the isocenter.

MO*Skin*™ measurements were acquired at WEDs of 0.07, 0.12, 0.52, and 1.07 mm using layers of 0.05 mm polyimide film and a 1.0 mm thick solid water sheet (Model number 457, Gammex/RMI, WI, USA) to compare the results from other datasets. Experimental ESE dosimetry beyond a depth of 1.0 mm was not explored in this work as the background photon tail region is already well‐documented.^5^, ^6^, ^12^, ^13^ Due to the finite lifespan of the MO*Skin*™, variable MUs were delivered to conserve each detector whilst ensuring a standard deviation of less than 2.0 cGy for three consecutive measurements. In line with previous studies,^5^, ^6^, ^12^, ^13^ MO*Skin*™ measurements were normalized to the maximum dose‐to‐water for a 10 × 10 cm^2^ field at an SSD of 133.5 cm.

The uncertainty of each normalized MO*Skin*™ dose value was determined using error propagation, with detailed information on the contributing components provided in Table S3. For each field size, two‐term exponential and spline functions were fitted to MO*Skin*™ measurements using the in‐built MATLAB (MathWorks Inc., MA, USA) function, “fit.” The exponential and spline fits were used to interpolate out‐of‐field MO*Skin*™ doses to equivalent WEDs of the other dosimeters and the center of the voxel size specified in the TPS.

In addition to interpolated MO*Skin*™ data, OSLD, film, and TPS‐reported doses were compared to the MO*Skin*™ measurements taken at a depth suitable for skin‐dose assessment. The relative difference, ΔD, between 0.07 mm WED MO*Skin*™ doses (D_ref_) and OSLD, film, and TPS doses, represented as D_x_, were calculated according to $\Delta {\;D}_{\left(x,\mathrm{ref}\right)}=\frac{(D_{x}-D_{\mathrm{ref}})\;}{D_{ref}}\;$. ΔD was expressed as a percentage (percent error) and used to assess the suitability of the MO*Skin*™, OSLDs, film, and TPS for out‐of‐field ESE skin dose assessment. The microDiamond was not included in this analysis as its design limits its use for in vivo dosimetry at surfaces nor can it measure depth doses shallower than 1.0 mm.

### 2.2 Optically stimulated luminescence dosimeters

OSLDs are solid‐state dosimeters that are commonly used for in vivo dosimetry. Commercial nanoDot OSLDs have been previously used to measure out‐of‐field electron streaming dose and are recommended for conventional out‐of‐field dosimetry by the vendor.^39^ The dose‐sensitive disk of the nanoDot OSLD is 5.0 mm in diameter and 0.3 mm thick, encapsulated in a hard‐plastic cassette (ρ = 1.0 g cm^−3^) measuring 10.0 mm × 10.0 mm × 2.0 mm.^39^ OSLDs have been used with the plastic case removed to eliminate the inherent build‐up^40^; however, we chose to follow vendor recommendations and had the disk contained within the cassette. The measurement point was assumed to be the center of the device and corresponds to a WED of 0.9 mm.^40^

#### 2.2.1 Calibration

For calibration, a group of nanoDot OSLDs from the same batch were positioned in a Solid Water® HE phantom that features custom‐machined recesses specifically designed for the OSLDs. The OSLDs were centered at isocenter, at a depth of 5.0 cm and an SSD of 138.5 cm. OSLDs used for calibration were individually irradiated with a 10 × 10 cm^2^, G0, beam with 50, 100, 300, 500, and 800 MU. OSLDs were then read out using the Landauer microStar reader (Landauer, Glenwood, IL, USA), and an average linear calibration factor, relating counts to dose, was determined for the batch.

#### 2.2.2 Measurements

For electron streaming measurements, the OSLDs were positioned at predetermined locations identified through RTQA2 film measurements. Per vendor recommendations, the visible raised cross‐hair on the surface of the OSLD plastic cassette^39^ was aligned to these locations. For each field size, two OSLDs were used to measure backscattered and ejected ESE with the same fields and setup geometry used for MO*Skin*™ measurements; however, 1000 MU was delivered for each field size. Doses were later read out using the microStar reader and normalized to the maximum dose (D_max_) deposited at d_max_ for a 10 × 10 cm^2^ field (SSD = 133.5 cm). The uncertainty of each OSLD dose value was determined using error propagation, with detailed information on the contributing components provided in Table S3.

### 2.3 Film

EBT3 film can provide high‐resolution 2D dose distributions and has been widely used for assessing OFDs from electron streams.^5^, ^6^, ^7^, ^8^, ^14^, ^15^, ^18^, ^22^ The WED of EBT3 film can be approximated to 0.14 mm which corresponds to the center of the active dose‐sensitive layer, set between the two substrate layers.

#### 2.3.1 Calibration

EBT3 film calibration was performed using 2.0 × 4.0 cm^2^ film strips within a 30.0 × 30.0 × 19.0 cm^3^ Solid Water® HE phantom. Films were positioned at isocenter, 5.0 cm depth within the phantom, and irradiated with a 10 × 10 cm^2^ G0 field and 0, 100, 200, 400, and 800 MU. The irradiated films were scanned and digitized using an Epson Expression 12000XL flatbed scanner (Seiko, Epson Corporation, Nagano, Japan) in transmission mode, without color correction, a scan resolution of 75 DPI, and using 48‐bit RGB mode. FilmQA™ Pro software (Ashland ISP Advanced Materials, NJ, USA) with RGB multichannel analysis^41^ was used for all film analysis. A central 1.0 × 1.0 cm^2^ region of interest (ROI) on each calibration film was used to correlate the mean pixel value with the delivered dose.

#### 2.3.2 Measurements

Films were positioned on the surfaces of the two outer solid water blocks, parallel to the x‐direction, to capture each electron stream dose distribution. Previous marks on RTQA2 film, which were used to position point dose detectors, were transposed onto each EBT3 film sheet. Backscattered and ejected ESE films were irradiated simultaneously, for each field size, with 1000 MU. Films were scanned as previously for calibration, and lateral scanner variations were corrected using a MATLAB script.^42^ Profiles across the ESE distribution in the x‐direction were created in FilmQA™ Pro using a 25.0 × 0.3 cm^2^ ROI. Along each profile, the dose corresponding to the position of the point‐dose dosimeter measurements was recorded. Film dosimetry used in this investigation followed a protocol that yielded a dose uncertainty below 3.0%.^42^ The uncertainty of each film dose value was determined using error propagation, with detailed information on the contributing components provided in Table S3.

### 2.4 Microdiamond detector

The PTW 60019 microDiamond (PTW, Freiburg, Germany) detector has previously been used for MR‐linac photon beam dosimetry.^34^, ^43^, ^44^, ^45^, ^46^ It is considered close to an ideal detector due to its near‐tissue equivalence, small sensitive volume (thickness 0.001 mm and diameter 2.2 mm), and water‐equivalent window thickness of 1.0 mm.^47^ As a result of its design, the microDiamond can produce excellent spatial resolution and is often used for small‐field dosimetry and shallow depth dose measurements where the minimum achievable WED is 1.0 mm.

#### 2.4.1 Calibration

The microDiamond detector used in this work was previously calibrated in terms of absorbed dose to water on the 7 MV Unity beam and a 1.054 nC/Gy calibration factor was obtained for a G0 10 × 10 cm^2^ beam at a measurement depth of 5.0 cm (SSD = 138.5 cm).

#### 2.4.2 Measurements

For ESE measurements, the microDiamond was positioned in a customized solid water block (Gammex Solid Water‐ Model # 457, Middleton, WI)^34^, ^35^, ^46^ phantom (Figure 2b). The microDiamond was connected to a Unidose electrometer (PTW, Freiburg, Germany) with a 0 V bias voltage, and the electrometer zeroed after 300 MU was delivered. A total of 150 MU was delivered three times for each field. Backscattered ESE and ejected ESE doses were measured separately. The average charge per MU was converted to dose and normalized to the dose at d_max_ for a 10 × 10 cm^2^ field (SSD = 133.5 cm). Vendor‐supplied documentation details a photon energy response uncertainty of ≤±4.0% for microDiamond use between 100 kV and ^60^Co.^47^ For scenarios where electron streams may form, the OFD is a combination of scattered primary photons and approximately 0.5 MeV electrons. For each measurement, the indicated microDiamond uncertainty was derived from the combined relative standard uncertainty, with the specific components outlined in Table S3.

## 3 TPS SIMULATED ESE DOSE

The Elekta Unity Monte Carlo‐based TPS, known as “Monaco” (V5.40, Elekta AB, Stockholm, Sweden), was used to simulate the measurement geometry. Monaco utilizes the graphical processing unit (GPU) Monte Carlo dose (GPUMCD) algorithm which enables fast dose calculations in the presence of magnetic fields.^6^

**FIGURE 3:**
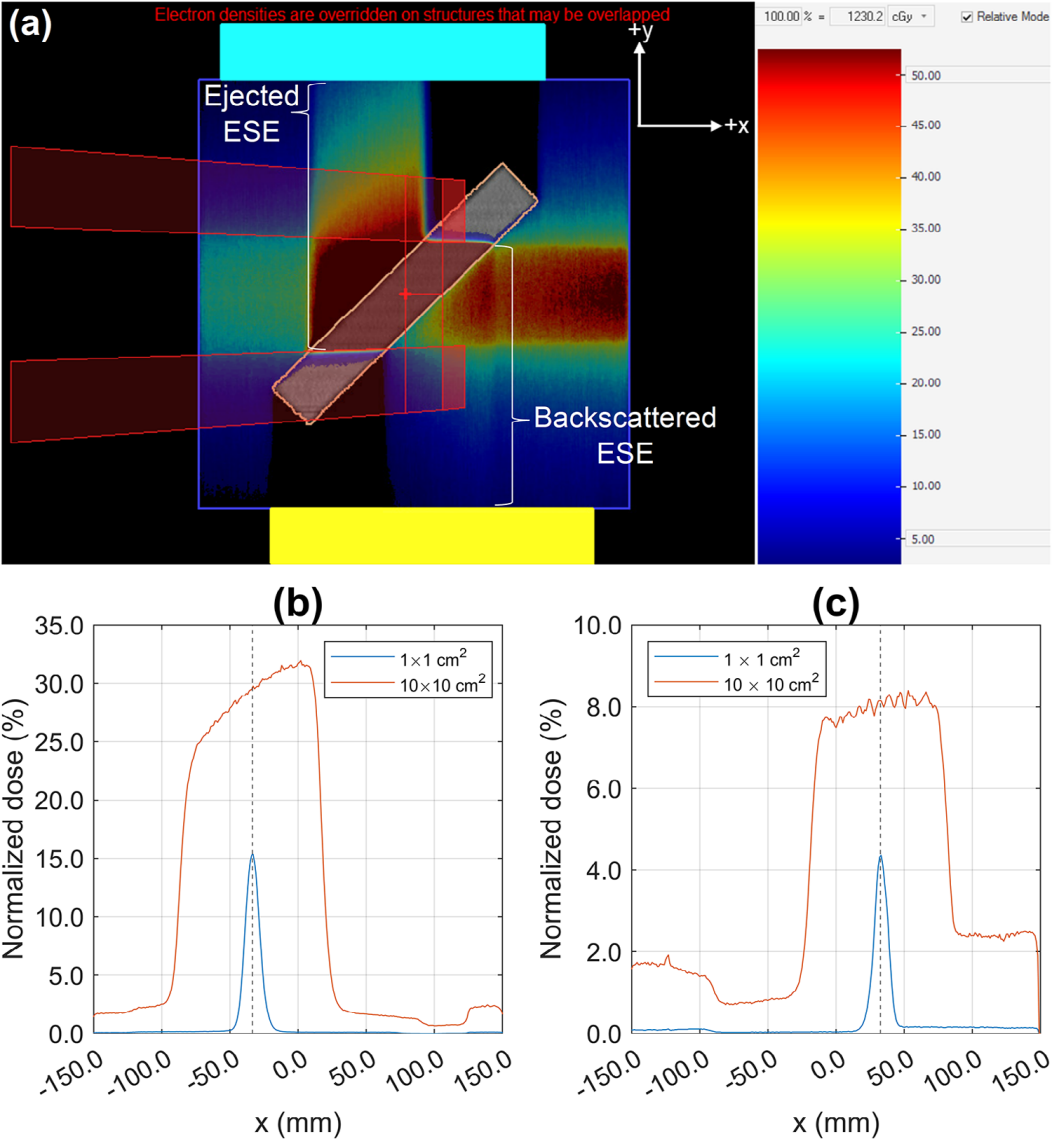
(a) A coronal slice of the calculated dose distribution for a G90, 1000 MU 10 × 10 cm^2^ beam irradiating the angled solid water. (b) Ejected ESE transverse dose profiles in the x‐direction for a 1 × 1 cm^2^ and 10 × 10 cm^2^ beam at the surface of the +y positioned solid water phantom. Similarly in (c) for backscattered ESE at the ‐y positioned block. The dashed vertical line in (b) and (c) indicates the position used for comparison between experimental and TPS computed datasets. All data is normalized to D_max_ of a 10 × 10 cm^2^ field.

To replicate the experimental geometry in the TPS, the 45° angled solid water phantom was outlined using a marker on the treatment couch and transposed onto paper. The setup was reproduced on a Toshiba Aquilion (Toshiba Medical Systems, Otawara, Japan) computer tomography (CT) couch, scanned with 1.0 mm slices, and imported into Monaco. The scanned phantom was contoured as an external structure, and additional contours coinciding with the out‐of‐field solid water “scoring” slabs were added. For dose calculations, the relative electron density (RED) of the central solid water and out‐of‐field blocks was forced to 1.00 (water). To visualize electron streams, the air surrounding the solid water blocks was contoured and the RED forced to 0.01.^6^ 1 × 1 cm^2^, 3 × 3 cm^2^, 5 × 5 cm^2^, 10 × 10 cm^2^, and 22 × 22 cm^2^ 1000 MU, G90, beams were added, with the isocenter coincident with the center of the contoured 45° block, as shown in Figure 3a. For comparisons to experimental data, unforced air RED was used for the TPS simulations.^7^

TPS simulations were performed using two different dose grid spacing sizes with varying statistical uncertainty (per control point). The first used a 0.1 cm grid spacing with 0.2% statistical uncertainty (0.1 cm, 0.2%) The second used a 0.2 cm grid spacing with 3.0% statistical uncertainty (0.2 cm, 3.0%), matching clinical settings used at the center where the experimental measurements were performed.^7^ The minimum user‐specified grid spacing and statistical uncertainty Monaco allows is 0.1 cm and 0.1% statistical uncertainty, respectively. A low statistical uncertainty is not often used as it requires a longer computational time for a larger number of particle histories. After the completion of each calculation, transverse dose planes at the surface of each out‐of‐field scoring block were exported from Monaco and imported into ImageJ software (National Institute of Health, Bethesda, MD, USA) to extract profiles. For the 0.1 cm, 0.2% and 0.2 cm, 3.0% dose calculations, dose planes at WEDs of 0.5 and 1.5 mm were extracted which correspond to the approximate center of the 1.0 and 2.0 mm scoring voxel layers, respectively. The dose from the second voxel layer, corresponding to a WED of 1.5 mm, for the 0.1 cm, 0.2% TPS calculations were also reported. For each field size and calculation setting, single‐pixel width (1.0 mm) profiles were extracted along the x‐axis and normalized to the maximum dose‐to‐water for a 10 × 10 cm^2^ field (SSD = 133.5 cm). A point dose along each profile was used to compare to experimental data. The coordinates of each point dose corresponded to the center of the 1 × 1 cm^2^ ESE dose distributions, as shown in Figure 3b,c.

## 4 RESULTS

**FIGURE 4:**
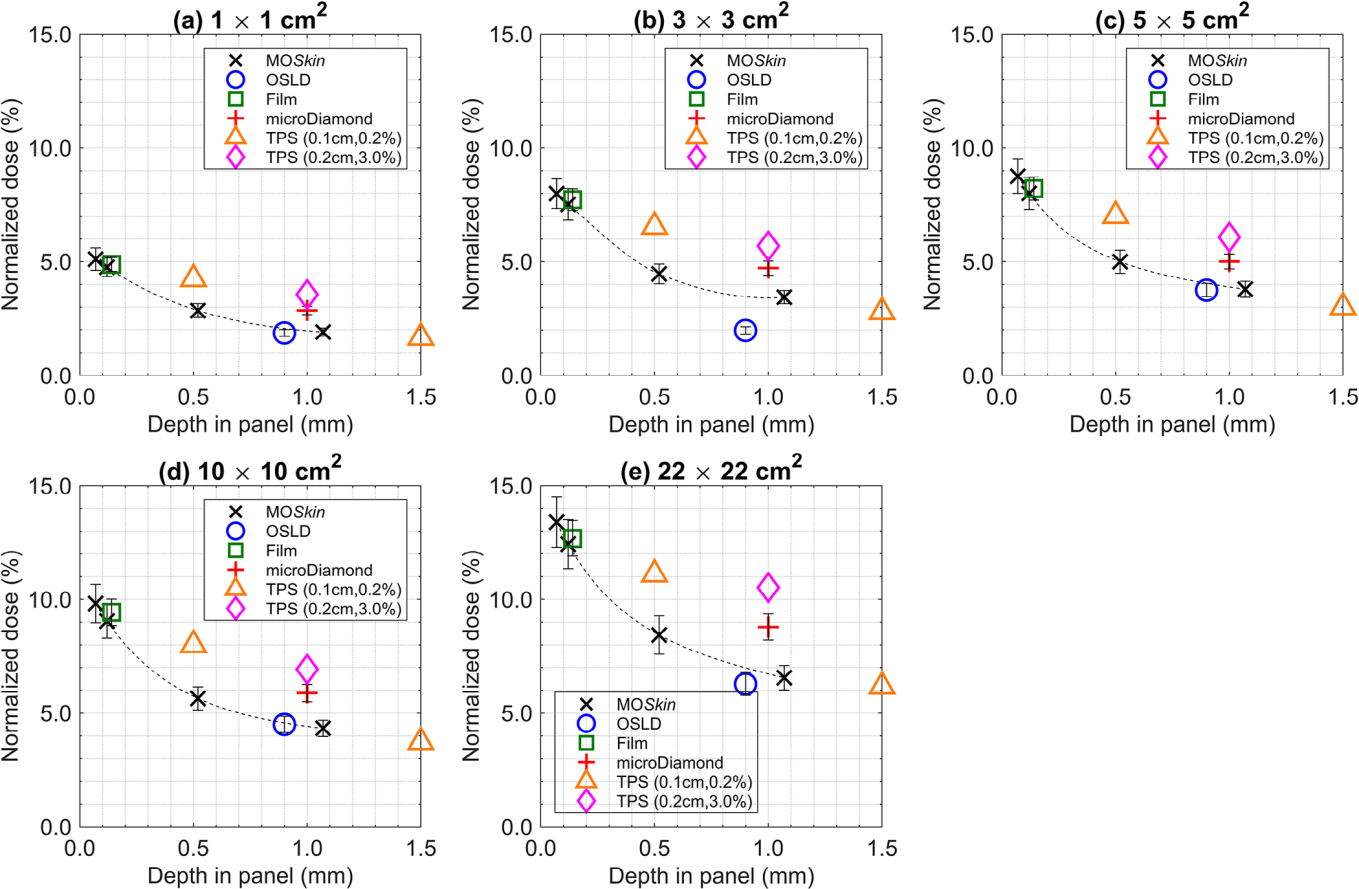
Measured and TPS predicted backscattered ESE doses for (a) 1 × 1 cm^2^, (b) 3 × 3 cm^2^, (c) 5 × 5 cm^2^, (d) 10 × 10 cm^2^, and (e) 22 × 22 cm^2^ fields. All data is normalized to Dmax of a 10 × 10 cm^2^ field. MOSkin™ (×), OSLD (○), film (□), microDiamond (+), TPS (0.1 cm, 0.2%) (△), and TPS (0.2 cm, 3.0%) (◇) point doses are denoted by their respective marker whilst the dashed line represents curve‐fit MOSkin™ doses. For each dosimeter measurement, the estimated error is given by an error bar.

**FIGURE 5:**
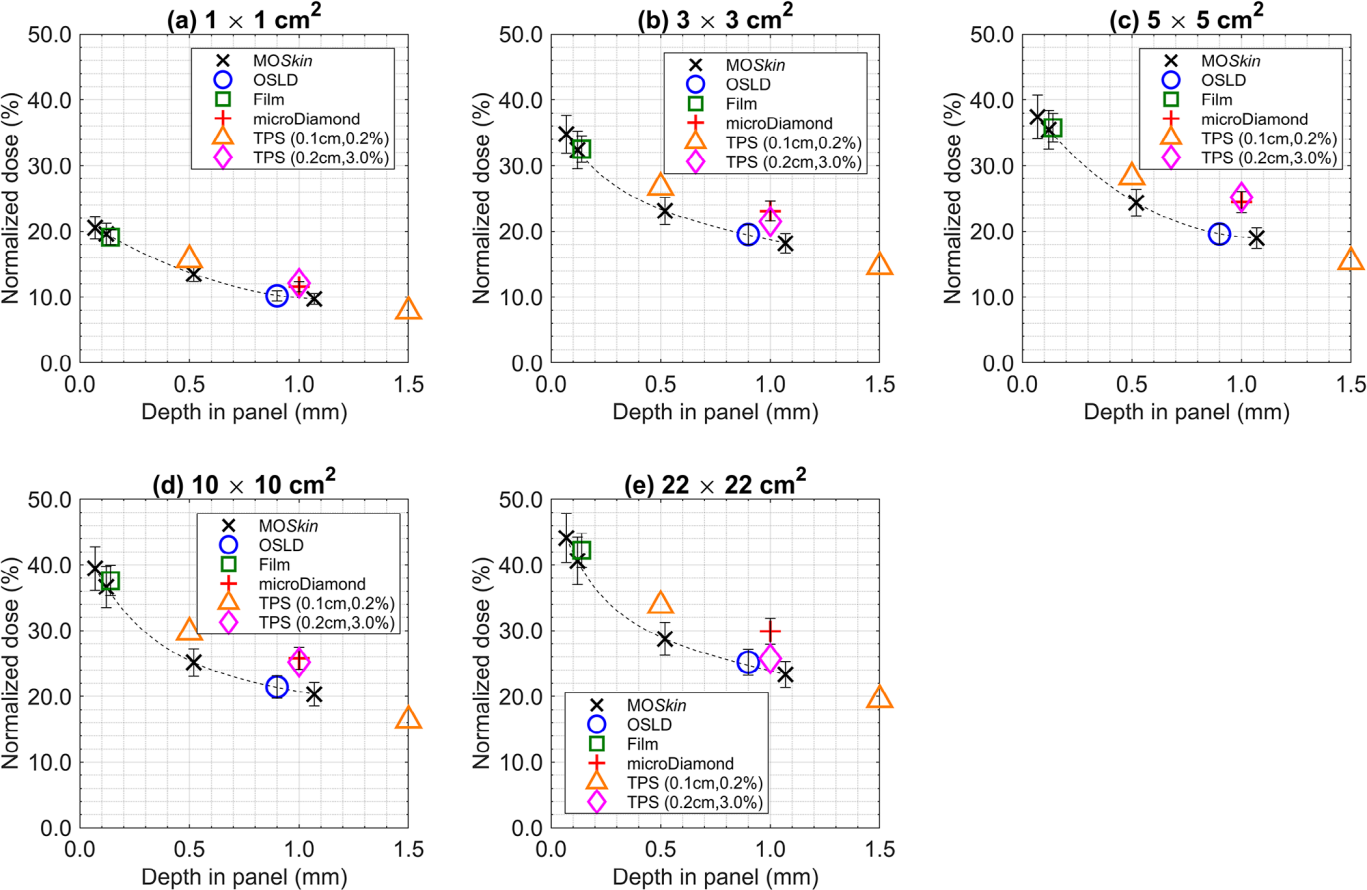
Measured and TPS predicted ejected ESE doses for (a) 1 × 1 cm^2^, (b) 3 × 3 cm^2^, (c) 5 × 5 cm^2^, (d) 10 × 10 cm^2^, and (e) 22 × 22 cm^2^ fields. All data is normalized to D_max_ of a 10 × 10 cm^2^ field. MOSkin™ (×), OSLD (○), film (□), microDiamond (+), TPS (0.1 cm, 0.2%) (△), and TPS (0.2 cm, 3.0%) (◇) point doses are denoted by their respective marker while the dashed line represents curve‐fit MOSkin™ doses. For each dosimeter measurement, the estimated error is given by an error bar.

Figures 4 and 5 display the experimental and the two distinct planning system dose calculation methods for backscattered and ejected electron stream doses, respectively. The continuous dashed lines in each figure represent curve‐fitted MO*Skin*™ data. The components contributing to the combined relative standard uncertainty of each dosimeter plotted as error bars in Figures 4 and 5, are described in depth in Table S3.

Using the 0.1 cm, 0.2% calculation parameters, the maximum statistical uncertainty of the ESE dose distributions in the TPS was 0.3%. For the 0.2 cm, 3.0% calculation settings, the statistical uncertainty of the TPS ESE dose distributions varied between 5.0% and 10.0% as reported by Monaco, depending on the field size and the origin of electrons (i.e., backscattered or ejected electrons).

All data is supplied in Tables S1 and S2. Two MO*Skin*™ detectors were used for experimental ESE dose measurements in this study, and the corresponding calibration factor for each detector was determined to be (2.31 ± 0.04) mV/cGy and (2.23 ± 0.02) mV/cGy, respectively.

MO*Skin*™ measured backscattered ESE doses at a 0.07 mm WED varied between (5.1 ± 0.5)% and (13.4 ± 1.1)%, relative to D_max_ for a 10 × 10 cm^2^ beam, over the range of fields investigated. Similarly, MO*Skin*™ measured doses of ejected ESE varied between (20.5 ± 1.7)% and (44.1 ± 3.7)%, relative to D_max_ for a 10 × 10 cm^2^ beam. All MO*Skin*™ measurements performed at a near‐equivalent WED of 0.12 mm were within experimental uncertainty to film doses. MO*Skin*™ measurements taken at a WED of 1.07 mm were consistent with OSLD readings, except for the 3 × 3 cm^2^ backscattered OSLD measurement, which fell outside the range of experimental uncertainty. As shown in Figures 4 and 5, MO*Skin*™ measurements acquired at a WED of 1.07 mm, fell outside the range of experimental uncertainty to microDiamond measurements across all fields investigated.

Except for the results obtained with the 22 × 22 cm^2^ field size, all MO*Skin*™ interpolated doses to a depth of 0.14 mm fell within the experimental uncertainty of film measurements performed in the ejected electron stream region. MO*Skin*™ doses interpolated to a depth of 0.9 mm, were mostly within experimental uncertainty to all OSLD measurements, except for the 3 × 3 cm^2^ and 22 × 22 cm^2^ backscattered ESE fields. For MO*Skin*™ doses interpolated to a WED of 1.0 mm, all values were outside the experimental uncertainty of the microDiamond measurements.

It was identified that TPS dose predictions acquired using 0.1 cm, 0.2% calculation properties were larger and outside experimental uncertainty of the MO*Skin*™ measurements taken at a WED of 0.52 mm for both backscattered and ejected electron streams. When interpolating MO*Skin*™ data, values calculated at 0.50 mm were within 0.4% of the reading acquired at 0.52 mm. For TPS calculations performed using the 0.2 cm, 3.0% settings, the estimated doses were outside experimental uncertainty to 1.07 mm WED MO*Skin*™ and microDiamond measurements for backscattered electron streams. Ejected ESE dose estimates using the 0.2 cm, 3.0% parameters generally fell between the curve‐fit MO*Skin*™ and microDiamond results. With field size changes it did not appear that the dose predicted by the TPS (0.2 cm, 3.0% plans) for ejected ESE consistently approached either the curve‐fit MO*Skin*™ data or microDiamond measurements.

**FIGURE 6:**
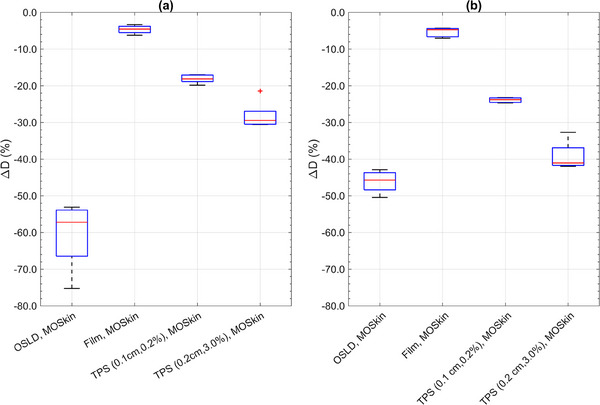
Box plot of the relative difference, ΔD, between the 0.07 mm WED MOSkin™ recorded dose values and OSLDs, film, and planning system values for (a) backscattered ESE, and (b) ejected ESE doses across all five field sizes investigated. For each box plot the red line indicates the median value, the box indicates the 25th–75th percentiles, the bar indicates extreme data points, and the red crosses indicate outliers.

Figure 6 illustrates the relative difference between the 0.07 mm WED MO*Skin*™ recorded values and OSLDs, film, and TPS‐reported doses at each of their respective measurement points for both streaming scenarios. As 0.07 mm is the recommended depth for skin dose assessment, 0.07 mm WED MO*Skin*™ doses were taken as the reference value for the relative difference calculations. Film data underreported skin dose in the range of −3.3% to −7.0% (relative difference) across all field sizes and electron streaming measurements. OSLD measurements underreported dose to the surface, suitable for skin dose assessment, between the ranges of −42.9% to −75.3% (relative difference) across all electron streaming measurements. For skin dose assessment, the TPS underreported OFD by as much as −24.7% and −41.9% (relative difference) for the calculation settings of 0.1, 0.2% and 0.2, 3.0%, respectively.

## 5 DISCUSSION

Variations in the dose from streaming electrons as a function of field size were quantified for a 7 MV FFF beam in the presence of a 1.5 T MRI unit using multiple dosimeters and the Monaco TPS.

MO*Skin*™ measurements suitable for skin dose assessment agreed within −7.0% (relative difference) to film data across all measurements. Poor agreement between the 0.9 mm WED OSLD and 0.07 mm WED MO*Skin*™ skin doses can be attributed to the attenuation of the secondary electrons in the external casing that surrounds the sensitive OSL material. Whilst removing the OSL material from the plastic cassette can reduce the dosimeters’ WED^40^ and potentially improve the accuracy of skin dose assessment, this method is not recommended by the vendor due to the light sensitivity of the OSL material and the possibility of mismatching an OSL element with its corresponding case, which lists the serial number and sensitivity specific to each device. Extrapolation of dose from at least two OSLDs with different WEDs positioned near the surface is another method that has been used to estimate skin dose^40^, ^48^; however, this can produce inaccurate results.^49^ The findings from this work indicate that EBT3 film is superior to the nanoDot OSLD for practical skin dose assessment from electron streams.

At a depth beyond the surface of the out‐of‐field phantom, the dose gradient of the streaming electron begins to flatten, resulting in an improved agreement between OSLD and interpolated MO*Skin*™ doses at the same WED. Whilst the majority of 0.9 mm MO*Skin*™ interpolated doses were within experimental uncertainty to OSLD measurements, the method, in general, has limitations particularly when the curve is determined using four experimental measurement points. The accuracy of the curve fit and therefore interpolated doses is contingent on the quality of the parameters used in the model. Based on a qualitative analysis of previous work by Malkov et al.,^13^ it was determined that the most appropriate model to describe the relationship between ESE attenuation and depth is an exponential regression model. Therefore, this model was chosen and used to interpolate MO*Skin*™ doses to different WEDs. For the 3 × 3 cm^2^ field size, backscattered ESE depth dose measurements of the MO*Skin*™ could not be properly fit using an exponential model, and a spline model was used instead. MO*Skin*™ interpolated doses were useful for comparing two dosimeters at equivalent WEDs; however, it is important to exercise caution when interpreting these findings.

While the out‐of‐field energy dependence of the nanoDot OSLD has been previously characterized in non‐magnetic environments,^50^, ^51^ its energy response in the context of electron streaming measurements is largely unknown, and investigation of such is beyond the scope of this work. The ratio between the mass collisional stopping power of aluminum oxide (the sensitive material of the nanoDot) and water can be used to estimate the differences in energy deposition.

Using the NIST database,^52^ the stopping power ratios of aluminum oxide to water were calculated across all available energies and normalized to one at 7 MeV. A maximum energy dependence of 2.7% was determined for the nanoDot between 350 keV and 7 MeV, where the lower energy level was estimated according to the CSDA range of electrons in the surrounding casing of the sensitive chip.

Similarly for the MO*Skin*™, the maximum variance in the ratio of silicon dioxide to water between 60 keV and 7 MeV was found to be 6.5%, taking into account the attenuation of electrons with energies up to 60 keV in the thin build‐up layer above the sensitive volume layer. Applying the same method, the maximum energy dependence variances for the film (NIST material: based on atomic composition by mass percent values^53^) and the microDiamond (NIST material: carbon) were 2.0% and 1.2%, respectively.

The combined relative standard uncertainty of each dose measurement took into account the relevant energy dependence of each device, positioning uncertainty, reproducibility of repeated measurements, uncertainties provided by the vendor, and the uncertainty related to the film handling protocol used, as detailed in Table S3. Using conservative estimates for both the energy dependence and positioning uncertainties, the outcome is an overall conservative combined relative standard uncertainty.

The microDiamond consistently exhibited larger OFDs across all field sizes compared to measurements taken with the MO*Skin*™ and OSLDs at nearby WEDs. Despite its low energy dependence, near tissue equivalence, small sensitive volume, suitability for small‐field dosimetry, and reproducible water‐equivalent window thickness, the microDiamond tended to overestimate the dose deposited by electron streams at a depth of 1.0 mm. It is worth pointing out that the reported doses from the film, microDiamond detector, and the planning system generally follow a linear pattern with depth. To our knowledge, the present study is the first to investigate out‐of‐field ESE doses using a microDiamond detector. The disagreement between MO*Skin*™ and microDiamond detector doses at 1.0 mm warrants further investigation, potentially using film, which is beyond the scope of the present work.

TPS simulations calculated with the 0.1 cm, 0.2% and 0.2 cm, 3.0% settings, predicted OFD to the surface of the phantom as large as 33.9% and 25.7%, respectively, whilst the corresponding MO*Skin*™ and film doses for the same irradiation conditions were (44.1 ± 3.7)% and (42.2 ± 2.6)%, respectively. For a similar geometry and field size, the backscattered dose simulated by Malkov et al. using EGSnrc Monte Carlo code^13^ was within 0.5% (difference in normalized doses) to the backscattered ESE dose predicted by the 0.1 cm, 0.2% TPS plan in this work. Due to different phantom thicknesses, which are known to largely dominate the maximum OFD from electron streams ejected from an angled phantom, comparisons between our ejected ESE data and Malkov et al.^13^ were not made. MO*Skin*™ measurements enabled ESE depth doses to be resolved at shallower depths than previous simulations and film measurements performed by Malkov et al.,^12^, ^13^ and further illustrate the steep dose gradient, particularly within the first millimeter, of streaming electrons when incident on a phantom.

Discrepancies between TPS‐predicted OFD and experimental measurements performed at the surface are consistent with the fact that TPS doses are averaged across each voxel in a heterogenous dose region while dosimeters such as the MO*Skin*™ and film have sensitive volumes that are sub‐millimeter in thickness. The accuracy of the TPS for skin dose can also be limited when the dose voxel layer assigned to the skin partially occupies air and the patient, leading to dose perturbations.^26^ The results from this study found that for a thin and angled phantom, the TPS underreported skin‐specific dose from electron streams by as much as −41.9% (relative difference), as shown in Figure 6b. Using a finer dose grid and smaller statistical uncertainty setting, the TPS consistently underreports skin dose by as much as −24.7% (relative difference). Whilst calculation parameters, such as the 0.1 cm, 0.2%, can be used to improve the accuracy of the TPS and reduce dose distribution uncertainties, practicality in the clinic is limited by extended computational times.^6^ Past studies have noted an overestimation of electron streaming dose calculated by the planning system.^15^, ^18^ However, drawing direct comparisons and reaching meaningful conclusions between the results of prior studies and this work is challenging, primarily due to differences in experimental setups and methods. These variations include the chosen dose assessment method (averaging across an area vs. point dose), the distance between the measurement location and field edges (where the out‐of‐field dosimetric accuracy of planning systems varies with distance from the field edge), and the clinical focus of the referenced studies.

Although the TPS has demonstrated its ability to accurately identify and model the overall shape of the dose distributions from electron streams,^6^, ^7^, ^12^ clinical staff should be aware of the predictable variance between the TPS and true skin dose in areas affected by electron streams. Nevertheless, discrepancies between measured surface doses and those predicted by the planning system become mostly academic provided sufficient bolus thickness (1 cm) is adopted to attenuate electron streams before they reach the patient surface.^8^ While in vivo OSLD measurements have been used to verify TPS‐predicted ESE doses outside the treatment fields,^24^ the results from our study promote the use of EBT3 film over the nanoDot for accurate skin dose assessment. The MO*Skin*™ readout system is currently incompatible for use during MR image sequences and consequently has yet to be used clinically in an MR‐linac. The Centre of Medical Radiation Physics (CMRP) at the University of Wollongong, Australia is actively working to modify the existing cable design to enable real‐time MO*Skin*™ dosimetry during MR imaging.

## 6 CONCLUSION

Several dosimeters were used in this work to measure backscattered and ejected electron streams generated from an angled phantom in a 7 MV MR‐linac. The dose gradient of deposited electrons and the different WEDs of each dosimeter used produced variable dose measurements for the same irradiation conditions. MO*Skin*™ measurements, suitable for ICRP‐recommended skin dose assessment, were compared to OSLD, film, microDiamond, and TPS‐predicted doses. The findings from this investigation demonstrate the limitations of classical dosimeters in measuring doses near the surface for regions impacted by electron streams. Benchmarked against the MO*Skin*™, EBT3 film dosimetry outperformed the nanoDot OSLD in terms of accurate skin‐specific dosimetry for electron streams. While the TPS was observed to consistently underreport skin dose, it remains a reliable indicator of the presence of electron streams. Identifying electron streams using the TPS should signal the need for implementing shielding, with the planning system guiding the positioning of such shielding methods. Consequently, we recommend that clinicians adopt a cautious approach when determining the necessity of electron stream shielding solely based on the doses predicted by the planning system. Additionally, the results of this study may be useful for Physicists when deciding on dosimeters to report OFD from streaming electrons.

## AUTHOR CONTRIBUTIONS

Elizabeth Patterson, Marcus Powers, Peter E. Metcalfe, Dean Cutajar, Bradley M. Oborn, and John A. Baines contributed to the preliminary investigation design involving experimental and simulated data. Elizabeth Patterson, Marcus Powers, Peter E. Metcalfe, Dean Cutajar, and John A. Baines contributed to the study's final conception and design. Experimental data collection was performed by Elizabeth Patterson, Marcus Powers, Peter E. Metcalfe, Dean Cutajar, and John A. Baines. Experimental analysis and Monaco treatment planning system (TPS) simulations were performed by Elizabeth Patterson and Marcus Powers. Elizabeth Patterson wrote the main body of the manuscript, with contributions to the final versions from Marcus Powers, Peter E. Metcalfe, Bradley M. Oborn, and John A. Baines. The final manuscript was reviewed and approved by all authors.

## CONFLICT OF INTEREST STATEMENT

D.Cutajar declares consulting with Electrogenics Laboratories Ltd which is commercializing the MO*Skin*™ detector. Elizabeth Patterson, Marcus Powers, Peter E. Metcalfe, Bradley M. Oborn, and John A. Baines have no relevant conflicts of interest to disclose.

## Supporting information

Supporting Information
