# Supplementary material for: Electron streaming dose measurements and calculations on a 1.5 T MR‐Linac
Source: J Appl Clin Med Phys. 2024 Apr 25;25(7):e14370. doi: 10.1002/acm2.14370 (PMC11244671; doi:10.1002/acm2.14370)
Supplement: Supplementary file 1 — Supporting Information [file ACM2-25-e14370-s001.docx]

# Supporting Information

## Data Tables

|  |  | Normalized dose (%) | | | | | |
| --- | --- | --- | --- | --- | --- | --- | --- |
|  |  | (1) | (2) | (3) | (4) | (5) | (6) |
| Field size  (cm^2^) | Depth | MO*Skin*™ | OSLD | Film | microDiamond | TPS  (0.1 cm,0.2%) | TPS  (0.2 cm,3.0%) |
| 1 × 1 | 0.07  0.12  0.14  0.50  0.52  0.90  1.00  1.07 | 5.1 ± 0.5  4.8 ± 0.4  4.6 (I)  2.9 (I)  2.8 ± 0.3  2.1 (I)  2.0 (I)  1.9 ± 0.2 | -  -  -  -  -  1.9 ± 0.2  -  - | -  -  4.9 ± 0.3  -  -  -  -  - | -  -  -  -  -  -  2.9 ± 0.2  - | -  -  -  4.2  -  -  - | -  -  -  -  -  -  3.6  - |
| 3 × 3 | 0.07  0.12  0.14  0.50  0.52  0.90  1.00  1.07 | 8.0 ± 0.7  7.5 ± 0.7  7.4 (I)  4.6 (I)  4.5 ± 0.4  3.5 (I)  3.4 (I)  3.4 ± 0.3 | -  -  -  -  -  2.0 ± 0.2  -  - | -  -  7.7 ± 0.5  -  -  -  -  - | -  -  -  -  -  -  4.7 ± 0.3  - | -  -  -  6.5  -  -  -  - | -  -  -  -  -  -  5.7  - |
| 5 × 5 | 0.07  0.12  0.14  0.50  0.52  0.90  1.00  1.07 | 8.8 ± 0.8  8.0 ± 0.7  7.7 (I)  5.1 (I)  5.0 ± 0.5  4.0 (I)  3.9 (I)  3.8 ± 0.4 | -  -  -  -  -  3.7 ± 0.3  -  - | -  -  8.2 ± 0.5  -  -  -  -  - | -  -  -  -  -  -  5.0 ± 0.3  - | -  -  -  7.0  -  -  -  - | -  -  -  -  -  -  6.1  - |
| 10 × 10 | 0.07  0.12  0.14  0.50  0.52  0.90  1.00  1.07 | 9.8 ± 0.8  9.0 ± 0.8  8.8 (I)  5.7 (I)  5.6 ± 0.5  4.6 (I)  4.4 (I)  4.3 ± 0.4 | -  -  -  -  -  4.5 ± 0.4  -  - | -  -  9.4 ± 0.6  -  -  -  -  - | -  -  -  -  -  -  5.9 ± 0.4  - | -  -  -  8.0  -  -  -  - | -  -  -  -  -  -  7.0  - |
| 22 × 22 | 0.07  0.12  0.14  0.50  0.52  0.90  1.00  1.07 | 13.4 ± 1.1  12.4 ± 1.1  12.1 (I)  8.5 (I)  8.4 ± 0.8  7.0 (I)  6.7 (I)  6.6 ± 0.5 | -  -  -  -  -  6.3 ± 0.5  -  - | -  -  12.7 ± 0.8  -  -  -  -  - | -  -  -  -  -  -  8.8 ± 0.6  - | -  -  -  11.1  -  -  -  - | -  -  -  -  -  -  10.5  - |

Supplementary Table S-1: Summary of the backscattered ESE doses measured using the MOSkin™, OSLDs, film, and the microDiamond when placed out-of-field at the surface of a solid water panel. TPS dose estimates obtained using different calculation parameters are reported in columns 5 and 6. The abbreviation (I), denotes interpolated results. All data is normalized to D_max_ of a 10 × 10cm^2^ field

|  |  | Normalized dose (%) | | | | | |
| --- | --- | --- | --- | --- | --- | --- | --- |
|  |  | (1) | (2) | (3) | (4) | (5) | (6) |
| Field size  (cm^2^) | Depth | MO*Skin*™ | OSLD | Film | microDiamond | TPS  (0.1 cm,0.2%) | TPS  (0.2 cm,3.0%) |
| 1 × 1 | 0.07  0.12  0.14  0.50  0.52  0.90  1.00  1.07 | 20.5 ± 1.7  19.6 ± 1.6  19.2 (I)  13.8 (I)  13.5 ± 1.1  10.3 (I)  9.9 (I)  9.7 ± 0.8 | -  -  -  -  -  10.2 ± 0.8  -  - | -  -  19.1 ± 1.2  -  -  -  -  - | -  -  -  -  -  -  11.6 ± 0.8  - | -  -  -  15.7  -  -  -  - | -  -  -  -  -  -  12.1  - |
| 3 × 3 | 0.07  0.12  0.14  0.50  0.52  0.90  1.00  1.07 | 34.8 ± 2.9  32.4 ± 2.8  31.6 (I)  23.4 (I)  23.1 ± 2.1  19.5 (I)  18.7 (I)  18.2 ± 1.5 | -  -  -  -  -  19.5 ± 1.5  -  - | -  -  32.5 ± 2.0  -  -  -  -  - | -  -  -  -  -  -  23.1 ± 1.5  - | -  -  -  26.7  -  -  -  - | -  -  -  -  -  -  21.5  - |
| 5 × 5 | 0.07  0.12  0.14  0.50  0.52  0.90  1.00  1.07 | 37.4 ± 3.3  35.5 ± 3.0  34.7 (I)  24.8 (I)  24.4 ± 2.0  19.7 (I)  19.2 (I)  19.0 ± 1.6 | -  -  -  -  -  19.6 ± 1.6  -  - | -  -  35.8 ± 2.2  -  -  -  -  - | -  -  -  -  -  -  24.5 ± 1.6  - | -  -  -  28.3  -  -  -  - | -  -  -  -  -  -  25.2  - |
| 10 × 10 | 0.07  0.12  0.14  0.50  0.52  0.90  1.00  1.07 | 39.5 ± 3.3  36.7 ± 3.1  35.7 (I)  25.5 (I)  25.1 ± 2.1  21.4 (I)  20.7 (I)  20.3 ± 1.8 | -  -  -  -  -  21.4 ± 1.7  -  - | -  -  37.6 ± 2.3  -  -  -  -  - | -  -  -  -  -  -  25.8 ± 1.7  - | -  -  -  29.7  -  -  -  - | -  -  -  -  -  -  22.9  - |
| 22 × 22 | 0.07  0.12  0.14  0.50  0.52  0.90  1.00  1.07 | 44.1 ± 3.7  40.6 ± 3.6  39.4 (I)  29.0 (I)  28.7 ± 2.5  24.7  23.9 (I)  23.3 ± 1.9 | -  -  -  -  -  25.2 ± 2.0  -  - | -  -  42.2 ± 2.6  -  -  -  -  - | -  -  -  -  -  -  29.9 ± 1.9  - | -  -  -  33.8  -  -  -  - | -  -  -  -  -  -  25.7  - |

Supplementary Table S-2: Summary of the ejected ESE doses measured using the MOSkin™, OSLDs, film, and the microDiamond when placed out-of-field at the surface of a solid water panel. TPS dose estimates obtained using different calculation parameters are reported in columns 5 and 6. The abbreviation (I), denotes interpolated results. All data is normalized to D_max_ of a 10 × 10cm^2^ field

## Propagation of uncertainty

|  | Dosimeter | | | |
| --- | --- | --- | --- | --- |
| Component of uncertainty | MOSkin™ | OSLD | Film | microDiamond |
| Type A  Reproducibility of repeated measurements (U_m_)^[[1]](#footnote-1)^ | Included | NA | NA | Included |
| Type B  Positioning of dosimeter at the correct position (U_pos_)^[[2]](#footnote-2)^  Approximate energy dependence of the detector to electrons (relative to water) (U_en_)^[[3]](#footnote-3)^  Uncertainty provided by the vendor or determined using the center’s clinical film protocol (U_vendor_)^[[4]](#footnote-4)^ | 5.0%  6.5%  NA | 5.0%  2.7%  5.5% | 5.0%  2.0%  3.0% | 5.0%  1.2%  4.0% |
| Combined relative standard uncertainty (U_c_) | $U_{C}=\sqrt{U_{m}^{2}+U_{pos}^{2}+U_{en}^{2}+U_{vendor}^{2}}$ | | | |

Supplementary Table S3: Combined relative standard uncertainty parameters for the experimental measurements.

## References

1. Berger MJ, Coursey JS, Zucker MA, Chang J. Stopping-Power & Range Tables for Electrons, Protons, and Helium Ions. *NIST Stand Ref Database 124*. Published online 2017.

2. Laudauer. microSTAR ii and nanoDots^TM^ Frequently Asked Questions about the implementation and use of microSTARii and nanoDots for accurately measuring dose in medical applications. Published online 2019. https://www.landauer.com/sites/default/files/50748 MicroStarii FAQ v8_Final_encrypted_.pdf

3. PTW Freiburg. *“Detectors Catalog”: Detectors for Ionizing Radiation, Including Codes of Practice*.; 2022. https://www.ptwdosimetry.com/en/products/microdiamond-detector

4. Van Der Walt M, Marsh L, Baines J, Gibson S, Shoobridge A, de Vine G. Performance evaluation of an LED flatbed scanner for triple channel film dosimetry with EBT3 and EBT-XD film. *Phys Eng Sci Med 2022 453*. 2022;45(3):901-914. doi:10.1007/S13246-022-01161-Z

1. The reproducibility of the MOSkin™ and microDiamond detectors was assessed via the maximum percentage uncertainty observed over three consecutive measurements (StdDev/mean). For single irradiation measurements, U_m_ is not available (NA). [↑](#footnote-ref-1)
2. Given a conservative set-up uncertainty in the cross-plane axis of 1 mm, the largest deviation of dose across all the measurements was estimated to be no larger than 5.0%, based on profiles obtained using the TPS and film measurements. [↑](#footnote-ref-2)
3. The mass collisional stopping power (MCSP) ratio of a material to water serves as an indication of the material’s relative response compared to water across a range of electron energies. For each dosimeter, the MCSP ratio across all energies provided by NIST^1^ was normalised to 1.0 at 7 MeV. The attenuation of low-energy electrons in the material surrounding the sensitive volume was taken into account and the maximum variation of the normalised MCSP ratio up to 7 MeV was used to determine each U_en_ value. [↑](#footnote-ref-3)
4. When available, the dose accuracy information provided by device vendors was integrated into the overall relative standard uncertainty value^2,3^. The film uncertainity was estimated at 3.0% in accordance with the center’s clinical film protocol.^4^ [↑](#footnote-ref-4)
